# Suitability of a diamine functionalized metal–organic framework for direct air capture†

**DOI:** 10.1039/d3sc02554c

**Published:** 2023-08-08

**Authors:** Saptasree Bose, Debabrata Sengupta, Christos D. Malliakas, Karam B. Idrees, Haomiao Xie, Xiaoliang Wang, Michael L. Barsoum, Nathaniel M. Barker, Vinayak P. Dravid, Timur Islamoglu, Omar K. Farha

**Affiliations:** a Department of Chemistry, Northwestern University 2145 Sheridan Road Evanston Illinois 60208 USA o-farha@northwestern.edu; b Department of Materials Science and Engineering 2220 Campus Drive, Room 2036 Evanston Illinois 60208 USA; c Department of Chemical and Biological Engineering, Northwestern University Evanston Illinois 60208 USA; d International Institute of Nanotechnology, Northwestern University Evanston Illinois 60208 USA

## Abstract

The increase in the atmospheric carbon dioxide level is a significant threat to our planet, and therefore the selective removal of CO_2_ from the air is a global concern. Metal–organic frameworks (MOFs) are a class of porous materials that have shown exciting potential as adsorbents for CO_2_ capture due to their high surface area and tunable properties. Among several implemented technologies, direct air capture (DAC) using MOFs is a promising strategy for achieving climate targets as it has the potential to actively reduce the atmospheric CO_2_ concentration to a safer levels. In this study, we investigate the stability and regeneration conditions of *N*,*N*′-dimethylethylenediamine (mmen) appended Mg_2_(dobpdc), a MOF with exceptional CO_2_ adsorption capacity from atmospheric air. We employed a series of systematic experiments including thermogravimetric analysis (TGA) coupled with Fourier transformed infrared (FTIR) and gas chromatography mass spectrometer (GCMS) (known as TGA-FTIR-GCMS), regeneration cycles at different conditions, control and accelerated aging experiments. We also quantified CO_2_ and H_2_O adsorption under humid CO_2_ using a combination of data from TGA-GCMS and coulometric Karl-Fischer titration techniques. The quantification of CO_2_ and H_2_O adsorption under humid conditions provides vital information for the design of real-world DAC systems. Our results demonstrate the stability and regeneration conditions of mmen appended Mg_2_(dobpdc). It is stable up to 50% relative humidity when the adsorption temperature varies from 25–40 °C and the best regeneration condition can be achieved at 120 °C under dynamic vacuum and at 150 °C under N_2_.

## Abstract 2

A comprehensive examination of mmen-Mg_2_(dobpdc) as a direct air capture adsorbent to determine its practical feasibility and performance for potential industrialization in mitigating climate change by reducing atmospheric CO_2_ levels.

## Graphical abstract

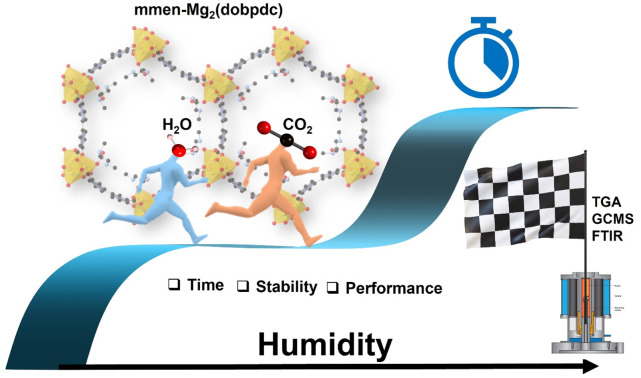

## Introduction

As the carbon dioxide (CO_2_) emissions continue to rise, climate change pushes the Earth towards an alarmingly dire situation. Reducing emissions will no longer be sufficient to mitigate this crisis, and we must establish facilities and governmental policies that can remove billions of tons of CO_2_ from the air annually.^1,2^ Among different methods of atmospheric CO_2_ reduction such as flue gas capture and other point source capture strategies, direct air capture (DAC) has shown promise in several literature and environmental reports for climate change moderation.^3,4^ DAC aims to extract CO_2_ selectively from ambient air where concentrations are as low as 400 ppm. Despite having the advantage that the capture point can be anywhere and recent favorable reports and successes of DAC technologies, finding an appropriate, reversible, cyclable, sorbent material for DAC of CO_2_ from dilute air (400 ppm of CO_2_) is still a major challenge.^5,6^ Selective adsorption of CO_2_ by liquid amine solution is one of the industrially viable options, but the high cost of regeneration, decomposition of amines with time, and its severe corrosive nature are major drawbacks for practical application in a DAC setup.^7,8^

**Fig. 1:**
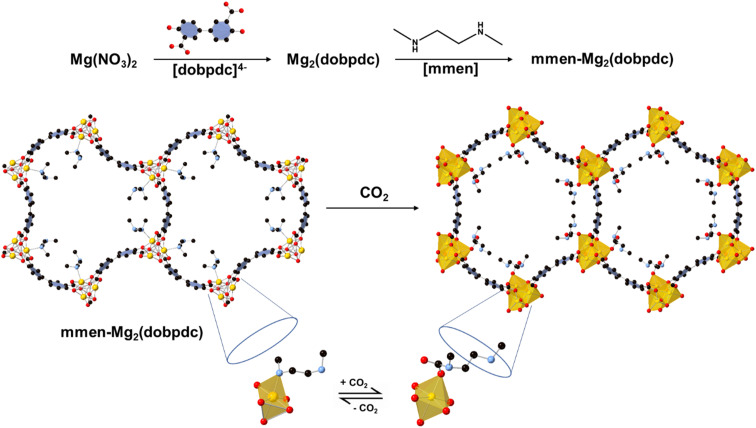
Schematic illustration of diamine (mmen) appended Mg_2_(dobpdc) synthesis and structure. The framework structure is adopted from single crystal X-ray analysis of the isostructural zinc compound DEF-1. Yellow, red, and gray spheres represent Mg, O, and C atoms respectively; H atoms are omitted for clarity. Addition of mmen to the unsaturated framework yields the amine-appended Mg_2_(dobpdc).^17,22^

Amine-functionalized solid porous adsorbents are an emerging class of materials which can selectively bind CO_2_ through carbamate formation. The strategy was first implemented with amine-grafted mesoporous silica for natural gas purification.^9,10^ Among other potential solid adsorbents, metal–organic frameworks (MOFs) have received growing attention due to their high degree of tunability, selectivity, and porosity (Fig. 1).^11^ Amine functionality within the ligand backbone has shown the possibility of selective capture of CO_2_ over water.^12^ However, total amine loading within the MOF pore makes the overall uptake fall short under real DAC conditions.^11,13^ Post-synthetic amine grafting, which attaches amines within the MOF pore, to an open metal site has improved the CO_2_ capture performance. In 2008, diamine grafting on the coordinatively unsaturated sites (CUS) of MOFs was first introduced by Férey and Chang *et al.* where the unsaturated Cr(iii) sites of MIL-101 are functionalized with ethylenediamine.^14^ Subsequently, in 2012 Jones *et al.* reported the first ethylenediamine (en) grafted Mg-MOF-74 (Mg_2_(dobdc), dobdc^4−^ = 2,5-dioxidoterephthalate) and explore the possibility of CO_2_ capture. The CO_2_ uptake capacity of en-Mg-MOF-74 is comparable to that of polyethyleneimide (PEI)-impregnated silica and amine grafted silica which is a well-known benchmark material.^15,16^ Mg-MOF-74 has unsaturated magnesium (Mg) centers that can act as Lewis acid and interact with guest molecules. Upon post-synthetic functionalization various diamines can be appended to an open metal site (OMS) in *κ*^1^ fashion leaving the other amine side free to interact with carbon dioxide. Long *et al.* have developed diamine appended pore expanded derivative of Mg-MOF-74 labeled as Mg_2_(dobpdc) (dobpdc^4−^ = 4,4′-dioxidobiphenyl-3,3′-dicarboxylate) which has an increased pore diameter (Mg-MOF-74: 11 Å *vs.* Mg_2_(dobpdc): 18.4 Å) and aids to tether more amine molecules within the channel.^17^ Ethylenediamine appended Mg_2_(dobpdc), named as en-Mg_2_(dobpdc) has capacity of capturing 2.83 mmol g^−1^ CO_2_ at 25 °C and 0.39 mbar or atmospheric CO_2_ levels, however a 6% loss of capacity was observed after first five cycles in simulated air. Long and others have tested various homo and heterodiamines to address the challenge of amine loss during regeneration to no avail.^18,19^ Among different homo and hetero diamines, *N*,*N*′-dimethylethylenediamine (mmen) appended Mg_2_(dobpdc) has been known to have high CO_2_ adsorption capacity at very low-pressure ranges with enhanced stability.^20^ A mechanistic study for CO_2_ adsorption in mmen-M_2_(dobpdc) (M = Mg, Co, Zn, Mn, Fe) has proposed that the adsorption of CO_2_ at ambient temperatures is possible *via* cooperative insertion into the mmen-M_2_(dobpdc) to form one dimensional carbamate chain and the uncoordinated amine of a mmen molecule acts as a strong base to remove the acidic proton from the metal-bound amine of a neighboring mmen molecule.^20^ Although the volatilization of coordinated diamine during the regeneration process upon dry and humid conditions remains unclear, some reports claim that diamine tethered Mg_2_(dobpdc) loses CO_2_ capture capacity due to a loss of diamine from the grafted sites.^18,21^

Therefore, it is essential to determine if the capacity fade is due to the degradation of the amine during the regeneration process or if the MOF has simply not been fully regenerated under standard conditions. Adsorption and desorption kinetics of CO_2_ have critical impact on the efficiency of the adsorbents, wherein the fastest kinetics allow for the highest cost effectiveness. mmen-Mg_2_(dobpdc) (Fig. 1) has been selected as a representative material system to understand the underlying chemical and structural changes that occur during the adsorption and desorption processes in relevant DAC conditions. Along with CO_2_ isotherms and cycling stability experiments, herein we identify additional metrics and experiments for evaluation of long-term performance to determine their applicability as DAC-sorbents. Real time thermogravimetric analysis (TGA) coupled with Fourier transformed infrared (FTIR) and gas chromatography mass spectrometer (GCMS) (known as TGA-FTIR-GCMS) allow us for deeper understanding of the mechanistic pathways of thermal regeneration. Since humidity is variable globally and temporally, it is important to evaluate the capture of CO_2_ in presence of water. Different experimental conditions were utilized to further understand the regeneration performance and suggest the desorption mechanism in dry and wet conditions. Moreover, we have performed 50 regeneration cycles under dry and humid conditions with different adsorption–desorption conditions along with long cycles test to assess their sorption performance. Finally, the capture of CO_2_ was quantified in presence of water by TGA-GCMS coupled instruments for determination of practical applicability of mmen-Mg_2_(dobpdc) globally.

## Results and discussions

### Synthesis, characterization, and stability of parent and diamine appended Mg_2_(dobpdc)

**Fig. 2:**
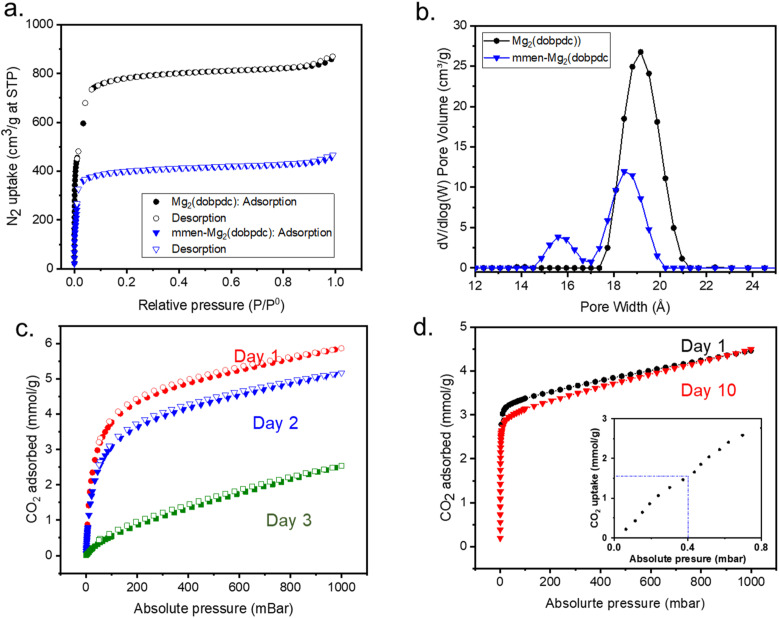
(a) N_2_ adsorption–desorption comparison isotherms of Mg_2_(dobpdc) (black) and mmen-Mg_2_(dobpdc) (blue) at 77 K. (b) Comparison of DFT pore size distribution of Mg_2_(dobpdc) (black) and mmen-Mg_2_(dobpdc) (blue). (c) CO_2_ uptake comparison isotherms of Mg_2_(dobpdc) with exposure time in air and at 25 °C and (d) CO_2_ uptake comparison isotherms of mmen-Mg_2_(dobpdc) with exposure time in air and at 25 °C (inset: low pressure CO_2_ uptake by mmen-Mg_2_(dobpdc)).

The phase purities of the mmen-Mg_2_(dobpdc) and diamine grafted MOF are aligned with simulated powder X-ray diffraction (PXRD) patterns. Moreover, crystallinity of the MOF is not affected after amine appendant as confirmed by PXRD measurement (Fig. S1†). The overall amine loading was confirmed by a ^1^H NMR study to be 50–60% amination per metal site (Fig. S2†). The pristine MOF and diamine-MOF show porosity and surface area in N_2_ isotherms at 77 K (Fig. 2a). Reduction in BET surface area and pore size for the amine functionalized MOF confirms the successful grafting of diamine to the metal sites of the MOFs. DFT pore size distributions and pore sizes were calculated from N_2_ adsorption at 77 K using the DFT pore model with cylinder pore geometries for an oxide surface. DFT pore size distributions show the amine appended structure has a smaller pore (Fig. 2b). BET surface areas for pristine MOF and mmen-MOF (60% amine per metal site) were calculated to be 2920 and 1550 m^2^ g^−1^, respectively.

Determination of the stability of the parent Mg_2_(dobpdc) and mmen-Mg_2_(dobpdc), was examined by exposing the samples to ambient conditions for several days while monitoring the CO_2_ uptake efficiency at 25 °C (Fig. 2c and d). The results indicate that the pristine MOF, Mg_2_(dobpdc) loses CO_2_ uptake capacity after three days, while the diamine appended MOF shows comparable CO_2_ uptake even after 10 days. The color of the pristine Mg_2_(dobpdc), which is linked to the phase, changes from off white to blue within 3 days of exposure to atmosphere whereas the off white color of mmen-Mg_2_(dobpdc) remains same even after 1 month. This additional stability from the diamine functionalization has been reported in literature.^17,23^ In addition to the increased stability the diamine appended MOF also shows stronger affinity towards CO_2_ at low pressure indicating involvement of chemisorption (Fig. 2d: inset).

### Mechanistic confirmation of carbamate formation monoamine grafted Mg_2_(dobpdc)

For a DAC application, adsorption capacity at 400 ppm is crucial since this mimic the concentration of CO_2_ under ambient conditions. Notably, the adsorption capacity of mmen-Mg_2_(dobpdc) at 400 ppm and 25 °C is 1.5 mmol g^−1^ (Fig. 2d (inset)). To validate the formation of carbamate during the capture of CO_2_, control experiments were performed to understand the role of diamine in the CO_2_ capture mechanism. Mg_2_(dobpdc) was functionalized with propylamine (pa) which is a monoamine following the same process used for diamine functionalization. PXRD patterns show the crystallinity is unchanged (Fig. S3†). ^1^H NMR studies confirmed the amine loading per metal site remains around 50–60% (Fig. S4†).

**Fig. 3:**
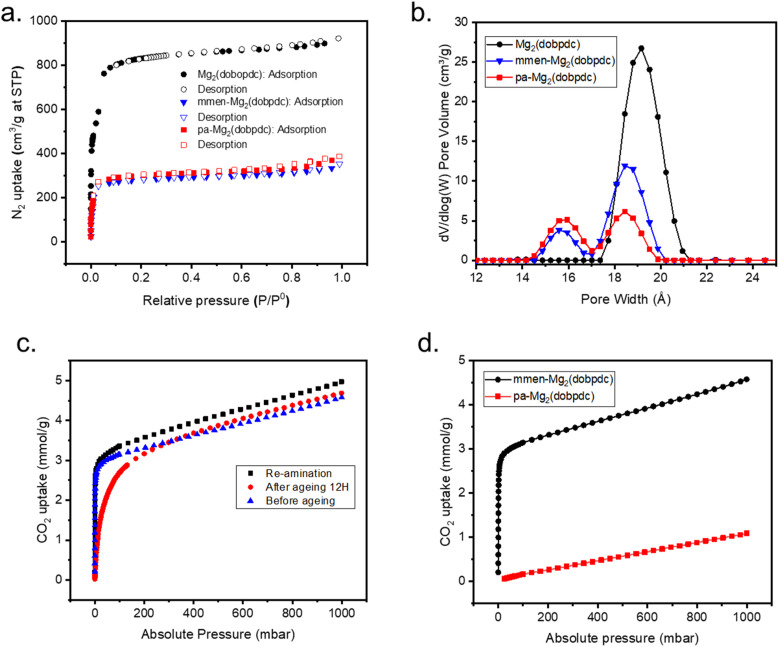
Control experiments (a) N_2_ isotherms of Mg_2_(dobpdc) (black), mmen-Mg_2_(dobpdc) (blue) and pa-Mg_2_(dobpdc) (red) at 77 K. (b) Pore size distributions of Mg_2_(dobpdc) (black), mmen-Mg_2_(dobpdc) (blue) and pa-Mg_2_(dobpdc) (red) (c) CO_2_ uptake of mmen-Mg_2_(dobpdc) (blue) and pa-Mg_2_(dobpdc) (red) at 25 °C; (d) CO_2_ uptake of mmen-Mg_2_(dobpdc) before ageing (purple), after ageing (orange), and after re-amination (green) at 25 °C. All the materials were used from the same batch.

The N_2_ isotherm shows BET surface area is compatible with mmen-Mg_2_(dobpdc) (Fig. 3a). Pore size distribution reveals the presence of two different pore sizes after functionalization with pa and is comparable with the pore size of mmen appended Mg_2_(dobpdc) (Fig. 3b). CO_2_ adsorption isotherm at 25 °C illustrates that the pa-Mg_2_(dobpdc) is not able to capture comparable amounts of CO_2_ as adsorbed by mmen-Mg_2_(dobpdc) and chemisorption part at lower pressure is also absent (Fig. 3c). These results indicate that presence of diamine is required to capture sufficient CO_2_ from atmosphere through the cooperative mechanism.^20^

Finally, the mmen-Mg_2_(dobpdc) was then aged through accelerated conditions at 80% relative humidity and 70 °C for 12 hours, ^1^H NMR indicates that upon exposure to such harsh conditions, amine loading was reduced from 60% to trace amount (Fig. S5†). Consequently, low-pressure (400 ppm) CO_2_ uptake is substantially decreased to 0.07 mmol g^−1^ from initial 1.5 mmol g^−1^ (for mmen-Mg_2_(dobpdc)) due to a reduction in the coordinated amines which is responsible for low pressure chemisorption (Fig. 3d). Interestingly, when the same sample was again re-aminated to 50–60% loading with the diamine the same CO_2_ uptake observed (Fig. 3d). This experiment confirms that the diamine is responsible for the chemisorption of CO_2_ and re-amination of the parent Mg_2_(dobpdc) is possible.

### Stability of coordinated diamine during regeneration process

**Fig. 4:**
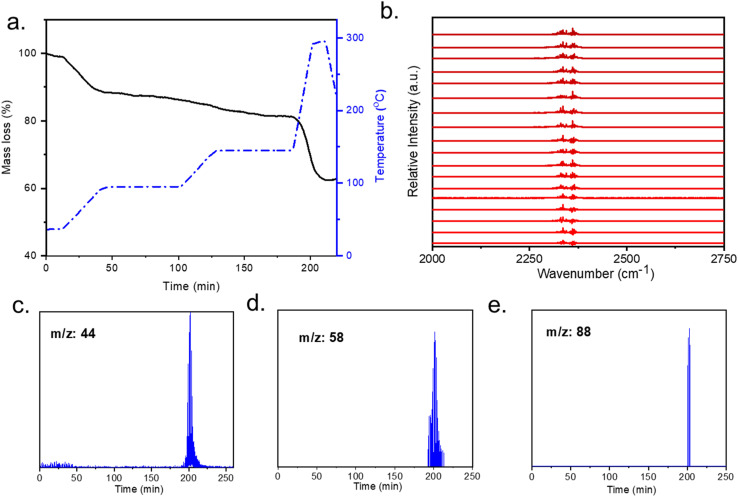
TGA-FTIR-GCMS experiments on mmen-Mg_2_(dobpdc) with adsorbed CO_2_: (a) thermogravimetric decomposition profile under humid He (black line); temperature ramp rate is shown in blue line; (b) FTIR spectra (0–50 min, 50–100 °C); characteristic CO_2_ was detected at 2360 and 2340 cm^−1^, each data was collected every 180 second. (c)–(e) gas chromatography mass spectra (GCMS) showing ions of released amine fragments.

mmen-Mg_2_(dobpdc) fully regenerates when heated at 100 °C for four hours under dynamic vacuum as confirmed by PXRD and N_2_ isotherms. Determination of the stability of the amines in the MOFs, was evaluated with thermogravimetric analysis (TGA) coupled with FTIR and GCMS. To verify the diamine stability in DAC conditions, it is also important to evaluate its thermal stability under humid conditions when it is fully adsorbed with CO_2_. The thermogram under humid He does not show any significant difference than that under dry He condition (Fig. 4a). CO_2_ (*m*/*z*: 44) is detected in GCMS at regeneration temperature 90–100 °C (Fig. 3c) which is due to the adsorbed CO_2_. The lack of amine leaching under the influence of moisture at this temperature indicates that the humidity does not affect the regeneration and stability of grafted amines. The CO_2_ signal can also be detected with FTIR measurements (Fig. 4b) during 50 minutes of thermal regeneration where the temperature was 50–100 °C indicating desorption of adsorbed CO_2_ at this temperature range. Fragmentations of diamine (*m*/*z*: 58, 88) are detected after 200 °C (Fig. 4d and e). The *m*/*z*: 44 detected after 200 °C (Fig. 4c) is attributed to the CO_2_ released for the degradation of diamine.

To determine conditions for the release of adsorbed CO_2_ the stability of diamine was evaluated with GCMS and FTIR spectra. Both spectra for the desorbed material show that the CO_2_ (*m*/*z*: 44) and fragmentations of diamine (*m*/*z*: 58 and 88) molecules are released only after 200 °C. The first one indicates the stability of adsorbent without any CO_2_ adsorption (Fig. S6†), which shows that until 200 °C, the diamine appended MOF is stable. There is no loss of coordinated diamine at the regeneration temperature. After a CO_2_ capture cycle the mmen-MOF also showed no amine peak by GCMS and FTIR spectra before 200 °C which indicates no loss of grafted amines during regeneration. CO_2_ molecules were released between 90-100 °C and after 200 °C (Fig. S7†). The first CO_2_ molecules detected around 90–100 °C are due to the desorbing of the CO_2_ in the host structure, while the CO_2_ molecules released after 200 °C are due to the degradation of the amines.

### Thermogravimetric CO_2_ adsorption–desorption under humid conditions

To establish a successful DAC experimental setup, understanding of the regeneration process of sorbents under dry and humid conditions is critical for establishing a protocol for DAC suitability. As demonstrated by TGA-FTIR GCMS no diamine loss occurs until 200–250 °C. However, the conditions for full desorption may illuminate the reason inherent capacity loss during cycling experiments.

**Fig. 5:**
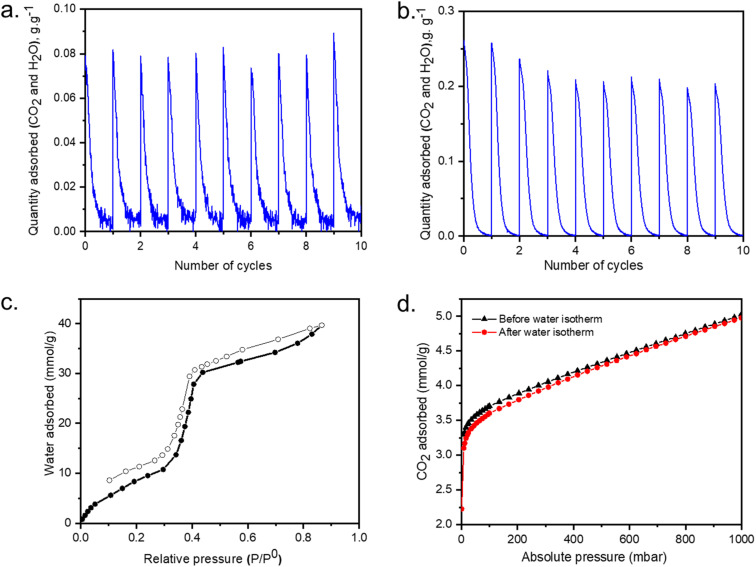
Regeneration performance cycles at 120 °C for mmen-Mg_2_(dobpdc): (a) after exposing to ambient air with 25% relative humidity; (b) after exposing to ambient air with 45% relative humidity; (c) water isotherm at 25 °C; (d) CO_2_ adsorption isotherms before and after measuring water isotherms at 25 °C.

The mmen-Mg_2_(dobpdc), was first regenerated and cycled at 120 °C for 30 minutes under N_2_ after exposing it to the ambient air. The desorption behavior under two humidity levels (25–30% RH and 40–50% RH) of the laboratory were assessed. After running 25–30% RH for 30 minutes (Fig. 5a) for 10 cycles (around 700 minutes), overall CO_2_ and/or H_2_O uptake from air was not decreased. But when the same cycle was performed after exposing to 45–50% RH (Fig. 5b), with 15% decrease in overall (CO_2_ + H_2_O) uptake was noticed.

Water isotherms were then collected at room temperature to assist in the understanding of water interactions within the structure. An S-shaped sorption isotherm was observed with the adsorbed amount of water gradually increasing until 30% RH, followed by an abrupt uptake between 40% and 50% RH, indicative of water filling into the pore system with the maximum uptake is 40 mmol g^−1^ at 90% RH (Fig. 5c). Water adsorption was found to be reversible with a small hysteresis over this material. Moreover, after activation at 120 °C under dynamic vacuum for 4 hours, CO_2_ uptake efficiency remained the same before and after water isotherm measurement (Fig. 5d).

TGA-GCMS of the mmen-Mg_2_(dobpdc), after exposing it to open environment (temperature: 27–32 °C, relative humidity: 30–50%) for several days, show that no amine leaching occurred at the regeneration temperature of 120 °C. However, CO_2_ and H_2_O are detected from the GCMS (Fig. S8 and S9†) indicating the diamine appended MOF adsorbs both CO_2_ and moisture from the open air.

### Assessing the impact of regeneration conditions on cycling stability

**Fig. 6:**
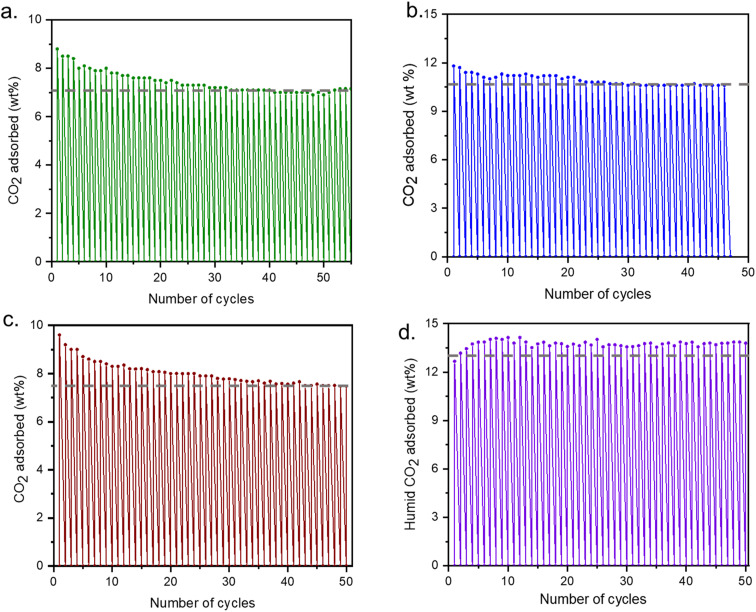
Adsorption–desorption cycling for mmen-Mg_2_(dobpdc): (a) desorption – CO_2_ at 150 °C (15 min) and adsorption – pure CO_2_ at 30 °C (15 min); (b) desorption – N_2_ at 150 °C (15 min) and adsorption – pure CO_2_ at 30 °C (15 min) (c) desorption – N_2_ at 150 °C (15 min) and adsorption – 15% CO_2_ in N_2_ at 30 °C (15 min) (d) desorption – N_2_ at 150 °C (15 min) and adsorption – humid 100% CO_2_ at 30 °C (15 min). The ramp rate was maintained as 10 °C for heating and cooling. The grey dashed line in each figure indicates the capacity of adsorbing dry and humid CO_2_ after 50 cycles.

Four different regeneration conditions (described in Fig. 6) were applied to determine if the cycling stability of the adsorbent is linked to the regeneration conditions of the sorbent (Fig. 6). It was found that the most optimal regeneration can be achieved under flowing N_2_ at 150 °C. In addition to these four conditions CO_2_ isobars with varying adsorption and desorption times as well as heating and cooling rates were also explored (Fig. S10–S13†). But it was found that time and ramp rate do not alter the total CO_2_ capacity of the adsorbent. For the long cycling experiments, we have maintained the adsorption and desorption time for 15 minutes for each case while the ramp rate was 10 °C min^−1^.

When desorption is carried out under CO_2_ atmosphere, 7% of CO_2_ capturing efficiency is maintained after 50 cycles (3000 minutes), but after 20 cycles it reduces to 7.5% from 9%, which means 80% of the materials capacity is retained (Fig. 6a). When the adsorbent was regenerated under N_2_ at 150 °C, 90% of the total capacity was maintained and almost no change in capacity was observed after 15–20 cycles (Fig. 6b). We have also performed the cycling experiment with 15% CO_2_ in N_2_ (resemble to flue gas) and 88% of total capacity is retained (Fig. 6c). Though no such decrease was observed after 20 cycles. So, the working capacity of the adsorbent under dry CO_2_, remains around 85–90% after 50 cycling experiments. Finally, under the humid cycle in which dry CO_2_ was passed through a water bubbler at room temperature before entering the sample chamber, there is no change in overall capacity and total (CO_2_ + H_2_O) capacity is maintained throughout the cycles (Fig. 6d). However, isolation of the relative concentration of each gas is needed to further determine the CO_2_ capacity.

### CO_2_ and H_2_O gas component quantification

**Fig. 7:**
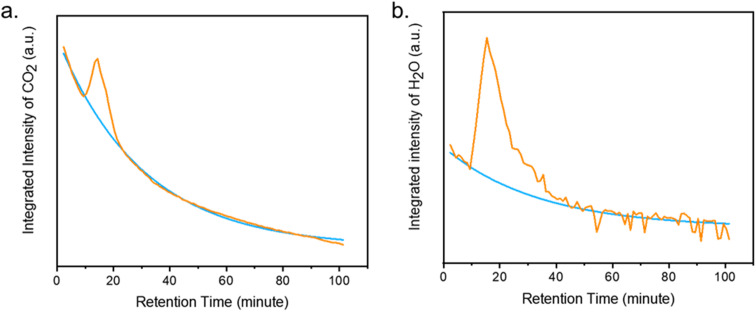
(a) Integrated intensity of desorbed CO_2_ (blue: background, orange: sample) and (b) integrated intensity of desorbed H_2_O (blue: background, orange: sample). Blue curve shows the background intensities of slow displacement of CO_2_ and H_2_O from the carrier gas (He), while the orange curve indicates the integrated intensity of the adsorbed CO_2_ and H_2_O from the chromatogram.

Despite best efforts to make sorbents selective to CO_2_, most adsorbents capture both CO_2_ and water. As we have seen, mmen-Mg_2_(dobpdc) captures both CO_2_ and H_2_O from air, as detected by TGA-GCMS during the regeneration of the MOF. Herein we report a technique to quantify the amount of CO_2_ and H_2_O using the coupled TGA-GCMS technique. Integrated intensities of chromatogram for CO_2_ (*m*/*z*: 44) and H_2_O (*m*/*z*: 18) were plotted against retention time of GCMS (Fig. 7a and b). Under humid CO_2_ (50% RH), the mmen-Mg_2_(dobpdc) adsorbed 1.7 mmol g^−1^ of CO_2_ (7.5% w/w) and 3 mmol g^−1^ of H_2_O (5.7% w/w) at 120 °C. Karl-Fischer coulometric titration on the sample (after re-exposure) corroborated that the sample uptakes around 3 mmol g^−1^ of water. Similar CO_2_ uptakes with other amine appended Mg_2_(dobpdc) and Mg_2_(dotpdc) (dotpdc^4−^ = 4,4′′-dioxido-[1,1′:4′,1′′-terphenyl]-3,3′′-dicarboxylate) have been reported by Long *et al.*^18,24^ The overall amount of adsorbed CO_2_+H_2_O is also consistent with the weight loss measured by TGA (Fig. S14†). The calibration curves for CO_2_ and H_2_O from standard calcium oxalate are shown in Fig. S15.† These values are significant to comment on the applicability of this system in real DAC plants.

## Conclusion

Amine appended-Mg_2_(dobpdc) is a well-known MOF which can capture CO_2_ at pressures as low as 400 ppm, making it a superior screening material for direct air capture. However, performance of these MOFs is dependent on the stability and recyclability under humid conditions. We have carried out a systematic study to assess the applicability of *N*,*N*′-dimethylethylenediamine (mmen) appended Mg_2_(dobpdc) MOF for direct air capture. With the help of coupled TGA-GCMS-FTIR, we have screened the performance of mmen-appended Mg_2_(dobpdc) under real DAC conditions. The quantification of exhaust gas revealed that this amine appended-Mg_2_(dobpdc) can uptake significant amount of CO_2_ (∼1.7 mmol g^−1^) in presence of 50% relative humidity. Regeneration is possible under appropriate conditions and can be enhanced through introduction of N_2_ gas under the regeneration conditions. These results have far-reaching implications that will afford the opportunity to engineer and design the process parameters like temperature, humidity, adsorption/regeneration conditions in similar diamine-appended MOF as future DAC sorbent.

## Data availability

Information supporting this article has been uploaded as part of the ESI.† Additional data is available from the authors on reasonable request.

## Author contributions

O. K. F. supervised the project. S. B., T. I., and O. K. F. conceived the project and led the investigation. S. B., D. S., T. I., and O. K. F. designed the experiments and interpreted the results, with help from K. I., H. X., and X. W. TGA-GCMS-FTIR characterization and controlled experiments were performed by S. B., C. D. M., and N. M. B. Manuscript was written by S. B., D. S., M. L. B., T. I., V. P. D., and O. F. K, and all other authors commented on and revised the manuscript.

## Conflicts of interest

The authors declare the following competing financial interest(s): O. K. F. has a financial interest in the start-up company NuMat Technologies, which is seeking to commercialize metal–organic frameworks.

## Supplementary Material

SC-014-D3SC02554C-s001
