# Supplementary material for: Suitability of a diamine functionalized metal–organic framework for direct air capture
Source: Chem Sci. 2023 Aug 8;14(35):9380–8. doi: 10.1039/d3sc02554c (PMC10498709; doi:10.1039/d3sc02554c)
Supplement: SC-014-D3SC02554C-s001 [file SC-014-D3SC02554C-s001.pdf]

Electronic Supplementary Material (ESI) for Chemical Science.
This journal is © The Royal Society of Chemistry 2023

Electronic Supplementary Information

Suitability of a Diamine Functionalized Metal-Organic Framework for Direct Air Capture

Saptasree Bose[a], Debabrata Sengupta[a], Christos D. Malliakas[a], Karam B Idrees[a], Haomiao Xie[a], Xiaoliang Wang[a], Michael L. Barsoum[b], Nathaniel M. Barker[a], Vinayak P. Dravid[b], Timur Islamoglu[a], Omar K Farha*[a,c,d]

[a] Department of Chemistry, Northwestern University, 2145 Sheridan Road, Evanston, Illinois 60208, United States

[b] Department of Materials Science and Engineering, 2220 Campus Drive, Room 2036 Evanston, Illinois, 60208, United States

[c] Department of Chemical and Biological Engineering, Northwestern University, Evanston, Illinois 60208, United States

[d] International Institute of Nanotechnology, Northwestern University, Evanston, Illinois 60208, United States

* Corresponding author: o-farha@northwestern.edu (Omar K. Farha)

Experimental Section

Materials: The linker, H_4(dobpdc) was commercially purchased. All other reagents and solvents were purchased from Sigma Aldrich and were used as received.

Synthesis of Mg_2(dobpdc) mmen- Mg_2(dobpdc), and pa-Mg_2(dobpdc)

Mg_2(dobpdc) was synthesized by following a reported procedure.[1] H_4(dobpdc) (27.4 mg, 0.10 mmol), $Mg(NO_3)_2{\cdot}6H_2O$ (64.0 mg, 0.25 mmol) and 10 ml of mixed solvent (55:45 methanol/dimethylformamide (DMF) were added into a 20 ml scintillation glass vial. The solid reagents were dissolved by sonication. The vial was sealed with a polytetrafluoroethylene (PTFE)-lined cap and the cap was resealed with Teflon tape and was placed in a heating block on a 120 °C hot plate. After heating for 12 hours, a white precipitate was formed on the bottom and walls of the vial. The white powder was collected after decanting off the reaction mixture and was soaked in DMF for three times with at least 2 hours intervals, and then soaked in methanol for three times with at least 2 hours intervals. Finally, the solid was collected by filtration and vacuum oven dried at 80 °C for 30 minutes and activated by heating under dynamic vacuum (<10 μbar) at 250 °C for 24 h.

mmen-Mg_2(dobpdc) was synthesized by a previously reported method.[2] Methanol-solvated Mg_2(dobpdc) was filtered and washed with toluene for 5 times and then it was submerged into excess solution of mmen in toluene (20% (v/v)). It was then soaked for 12 hours, and the solid was collected by filtration and washed with excess toluene to remove excess diamine. After vacuum oven drying for 30 min at 80 °C, the sample was activated under dynamic vacuum (<10 μbar) at 100 °C for 10 hours.

Propylamine (pa) modified pa-Mg_2(dobpdc) was synthesized and characterized by the procedure outlined above but mmen was replaced with pa during the synthesis.

Materials Characterization

Gas sorption measurements

N_2 physisorption isotherms of MOFs were measured at 77 K using a Micromeritics ASAP 2420 surface analyzer. DFT pore size distributions were calculated from N_2 adsorption at 77 K using a kernel for a metal oxide surface with a cylinder pore geometry. CO_2 and water isotherms at room temperature were measured with Micromeritics 3Flex. Prior to the water adsorption measurements, water (analyte) was flash frozen under liquid nitrogen and then evacuated under dynamic vacuum at least three times to remove any dissolved gases in the water reservoir. The temperature for water isotherm was controlled with a Micromeritics ISO Controller.

Powder X-Ray Diffraction (PXRD) and Nuclear Magnetic Resonance (NMR)

The PXRD data were collected on a STOE-STADI P powder diffractometer operating at 40 kV voltage and 40 mA current with Cu-Kα1 X-ray radiation (λ = 0.154056 nm) in transmission geometry. The instrument was calibrated against a NIST silicon standard (640d) prior to the measurements. Finally, ^1H NMR was performed using Bruker Avance HD 500 MHz w/Prodigy probe.

Thermogravimetric analysis (TGA)

mmen-Mg_2(dobpdc) samples were carefully transferred to TGA for thermal stability and CO_2 adsorption experiments with minimum exposure to ambient conditions. Dry thermogravimetric analysis (TGA) experiments were conducted using Discovery TGA5500 (TA Instruments, New Castle, USA). Humid TGA experiments were conducted using Netzsch STA 449 F3 “Jupiter” simultaneous thermal analyzer. For humid experiments, the incident carrier gas (helium) stream was passed through one room-temperature water bubbler before entering to the sample chamber. By using the August-Roche-Magnus approximation, the relative humidity in the stream was ~50% as the temperature of the room was 23 ˚C with a dew point of 12 ˚C. Before doing the thermal stability experiment, samples were activated under dynamic vacuum at 120 °C for four hours. For CO_2 adsorption experiments the samples were first activated under dynamic vacuum and then activated under flowing N_2 at 120 °C for 30 min or 60 min. Before the

gas uptake experiment, the temperature was cooled to the 30 °C and the gas was switched to 100% CO_2. A flow rate of 25 mL/min was used for all TGA experiments. Ramp rates for all isobaric measurements are described within figure captions.

TGA-FTIR-GCMS: experiments were performed using Netzsch STA 449 F3 “Jupiter” simultaneous thermal analyzer, a Bruker Tensor 37 spectrometer with a ZnSe crystal ATR attachment as infrared spectrometer, and Agilent 5973 GCMS. The instruments are coupled such that the emitted gases from sample in TGA instrument are exhausted into two lines: The first line is connected to the FTIR instrument and the second to the GCMS instrument. FTIR is triggered at the beginning of the TGA experiment while the GCMS is triggered at the predefined intervals during the TGA temperature profile.

Quantification of gases

To quantify the exhaust CO_2 and H_2O gasses in the TGA GC-MS was used. A calibration curve was made using certified calcium oxalate (European Pharmacopoeia Reference Standard, code: C0350000) as a source of CO_2 and H_2O. The same GC-MS method was utilized for all measurements of calcium oxalate and samples. During heating of the samples in the TGA, a gas injection was triggered every minute by an automated gas valve. The area of the same number of peaks/injections was integrated for the quantification. Coulometric Karl-Fischer titration was performed at room temperature using a Mettler-Toledo C30 titrator. A stream of released H_2O was transferred into the coulometric cell which was filled with a methanol-based electrolyte (Aquastar CombiCoulomat fritless) and equipped with a double Pt sensor. Absorbed H_2O was released with the aid of a drying oven attachment (D0308) on the titrator under a continuous flow of ultra-high purity dry nitrogen flow. The oven temperature was maintained at 120 °C.

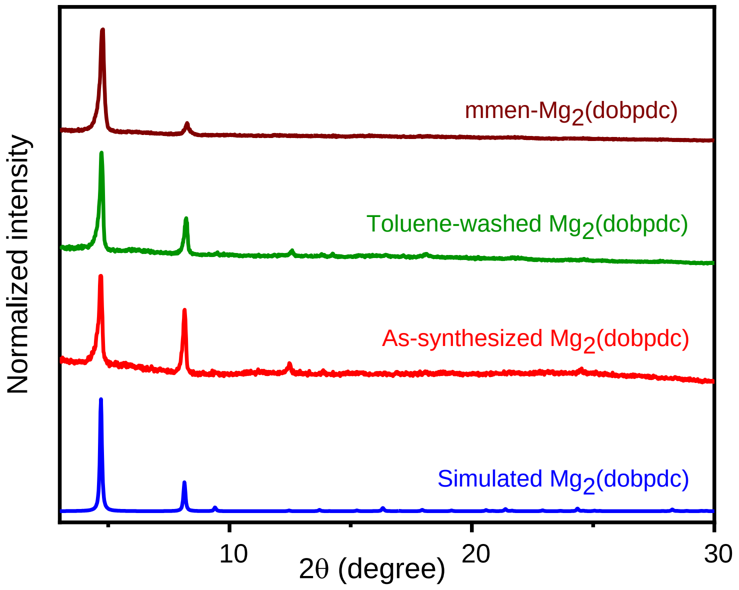

Fig. S1. Experimental X-ray powder diffraction patterns of simulated Mg_2(dobpdc) (blue), as-synthesized Mg_2(dobpdc) (red), toluene-washed Mg_2(dobpdc) (green), and diamine (mmen) functionalized Mg_2(dobpdc) (wine)

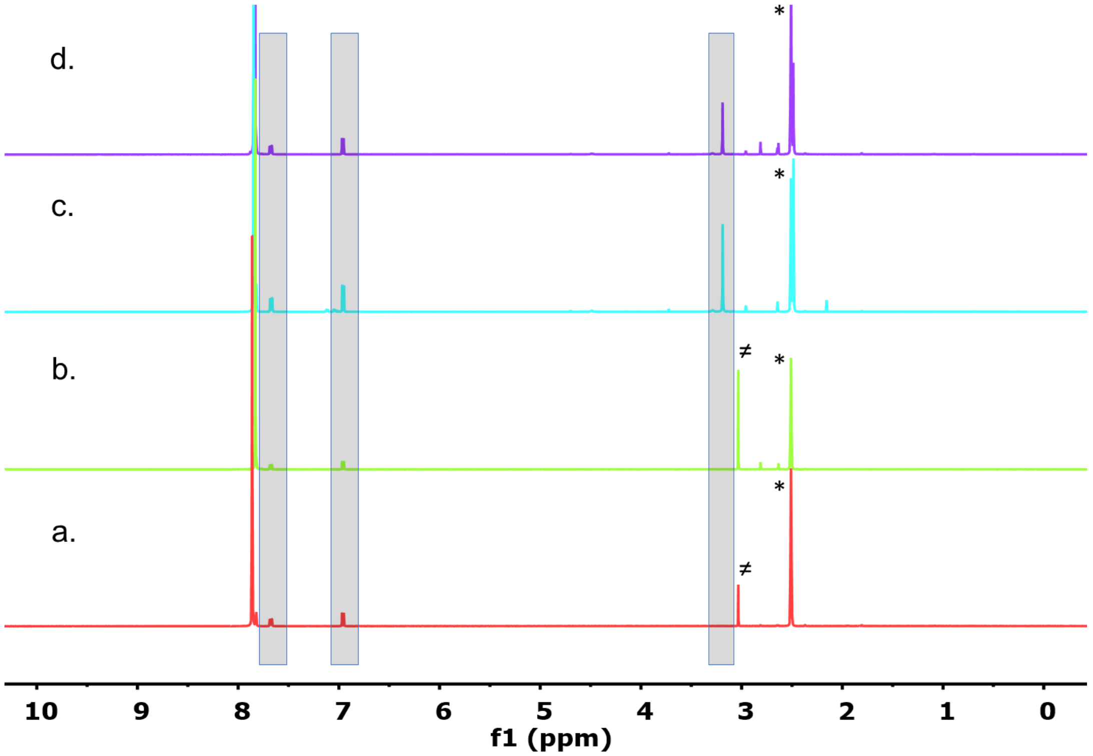

Fig. S2. ^{1}H NMR spectra in dmso-D_6/DCl: for a. Mg_2(dobpdc)-as synthesized; b. Mg_2(dobpdc)-MeOH washed; c. mmen-Mg_2(dobpdc)-as synthesized; d. mmen-Mg_2(dobpdc)-activated. The linker and the mmen are shown as shaded. These are used for calculating % of amine loading. Residual solvent peak (*), residual methanol peak(#).

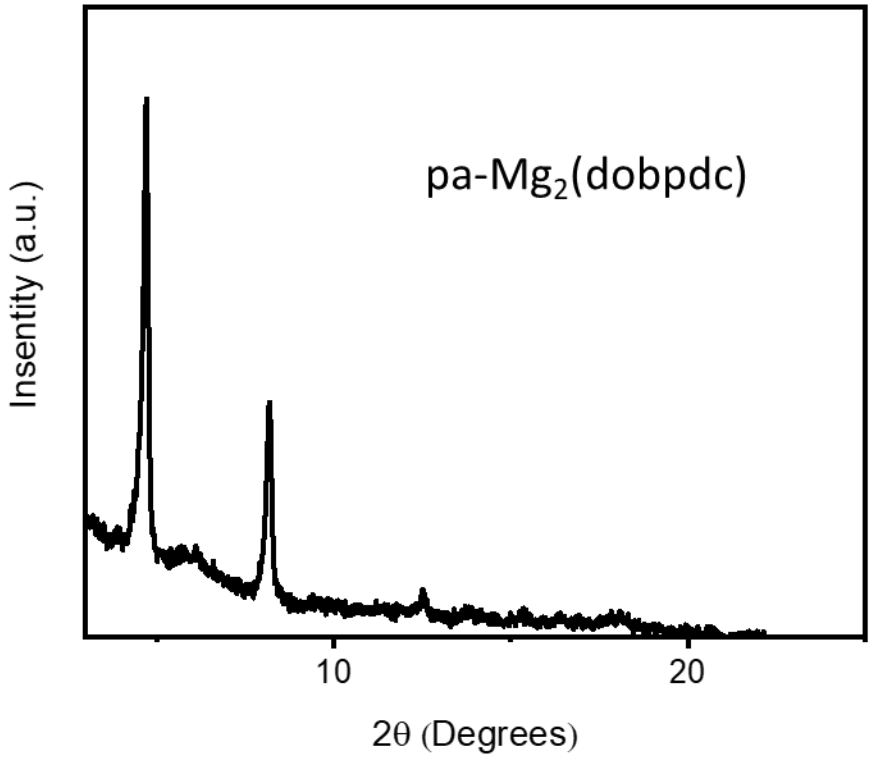

Fig. S3. Experimental X-ray powder diffraction patterns of PA-Mg_2(dobpdc)

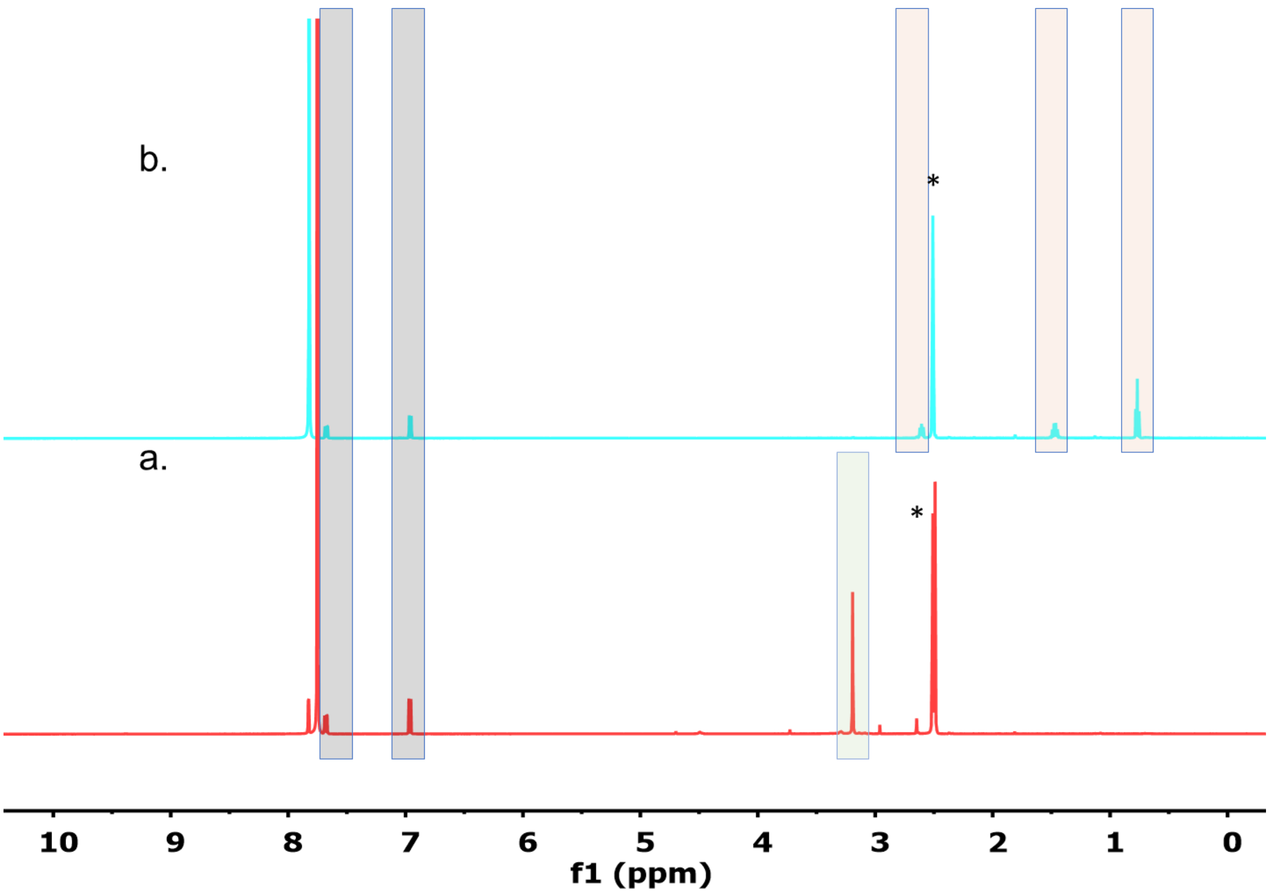

Fig. S4. ^{1}H NMR spectra in dmso-D_6/DCl: a. mmen-Mg_2(dobpdc) and b. PA-Mg_2(dobpdc). The linker (grey), mmen (green) and PA (orange) are shown as shaded. These are used for calculating % of amine loading. Residual solvent peak (*).

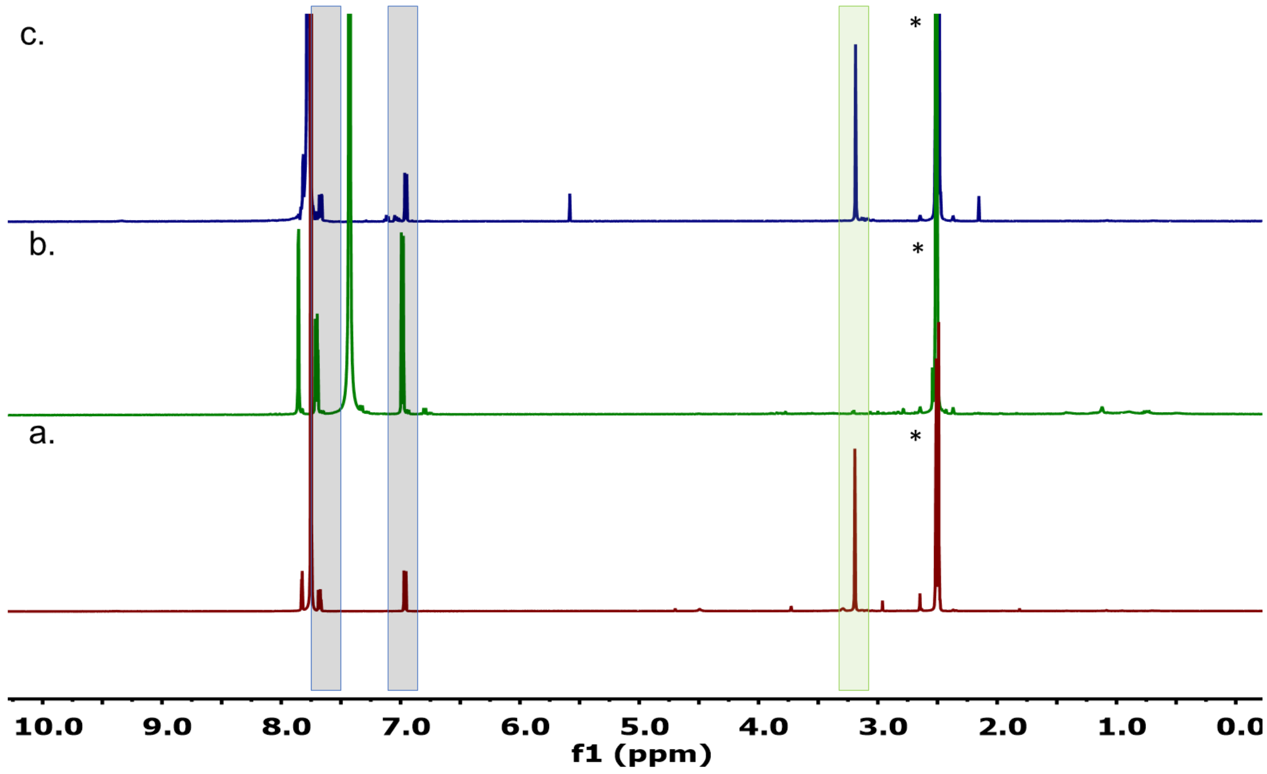

Fig. S5. ^1H NMR of mmen-Mg_2(dobpdc): a. before aging, b. after aging, and c. after re-amination of the aged sample. The linker (grey) and the mmen (green) are shown as shaded. These are used for calculating % of amine loading. Residual solvent peak (*).

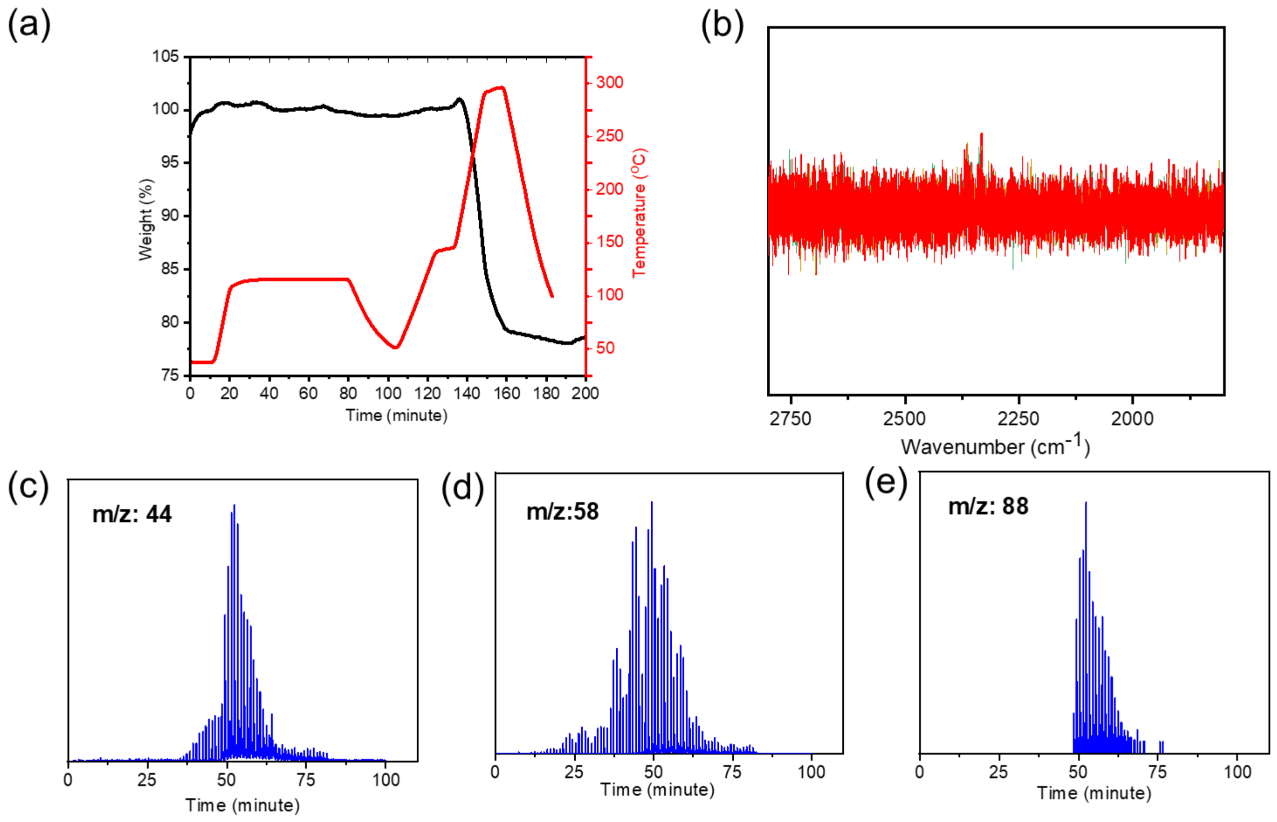

Fig. S6. TGA-FTIR-GCMS experiments on mmen-Mg_2(dobpdc) without CO_2 adsorption: (a) Dry He decomposition profile (black solid line) and temperature ramp rate is shown in red solid line; (b) FTIR spectra: characteristic CO_2 was detected at 2360 and 2340 cm^{-1} (c); (d); and (e) gas chromatography mass spectra (GCMS) of amine fragments released at 200 ^{o}C.

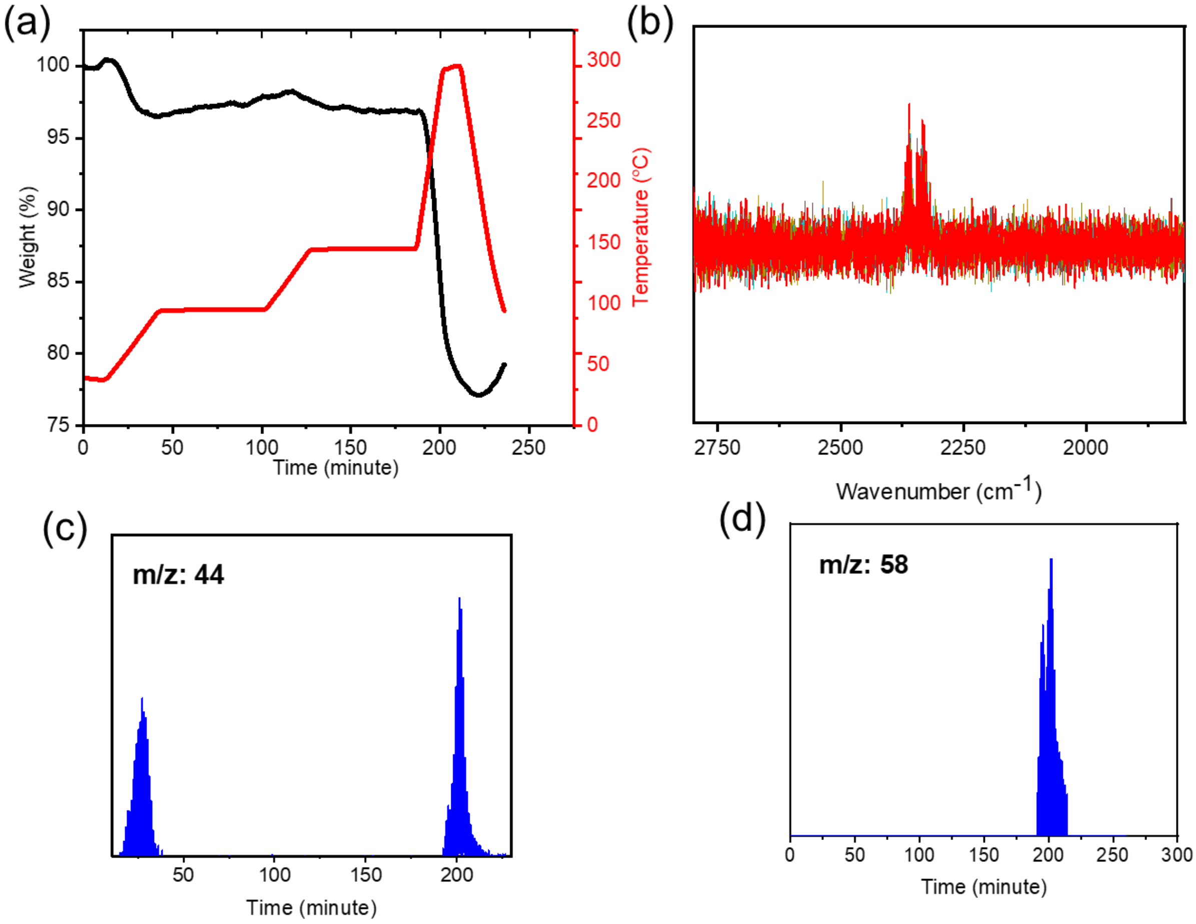

Fig. S7. TGA-FTIR-GCMS experiments on mmen-Mg_2(dobpdc) adsorbed with CO_2: (a) Dry He decomposition profile (black solid line) and temperature ramp rate is shown in red solid line; (b) FTIR spectra; characteristic CO_2 was detected at 2360 and 2340 cm^{-1}. Gas chromatography mass spectra (GCMS) showing ions of released gases: (c) CO_2 and (d) amine fragments.

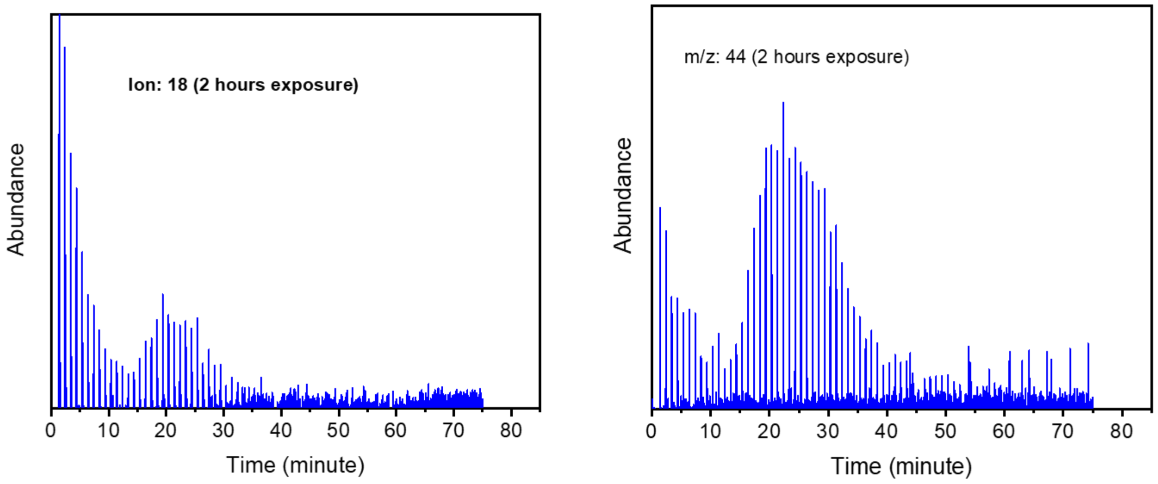

Fig. S8. Gas chromatography mass spectra (GCMS) during regeneration at 120°C showing H_2O and CO_2 ions (m/z: 18 and 44) after exposing the adsorbent to indoor humidity (30-40%) at room temperature for two hour.

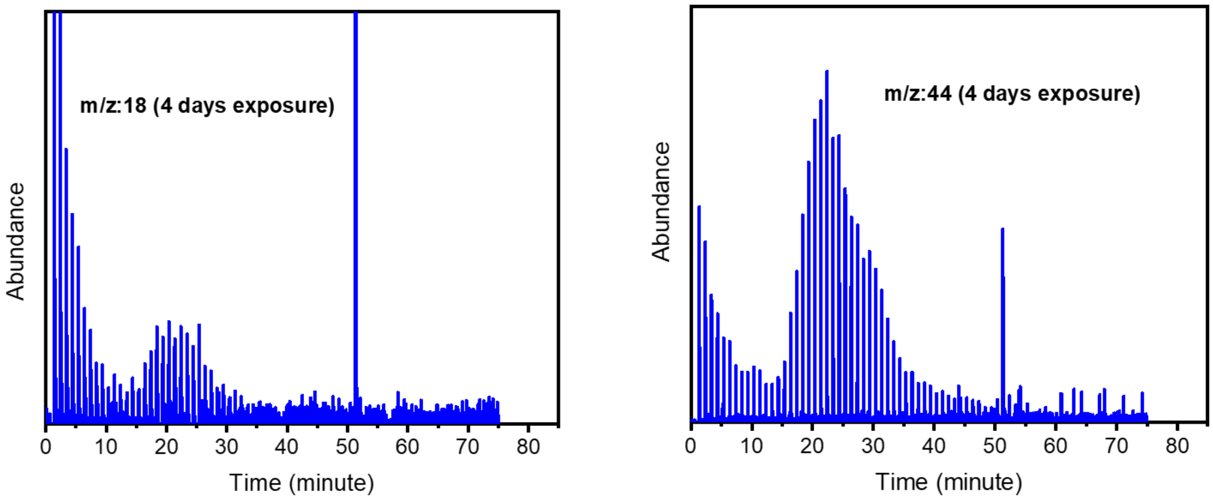

Fig. S9. Gas chromatography mass spectra (GCMS) during regeneration at 120°C showing H_2O and CO_2 ions (m/z: 18 and 44) after exposing the adsorbent to indoor humidity (30-40%) at room temperature for 4 days.

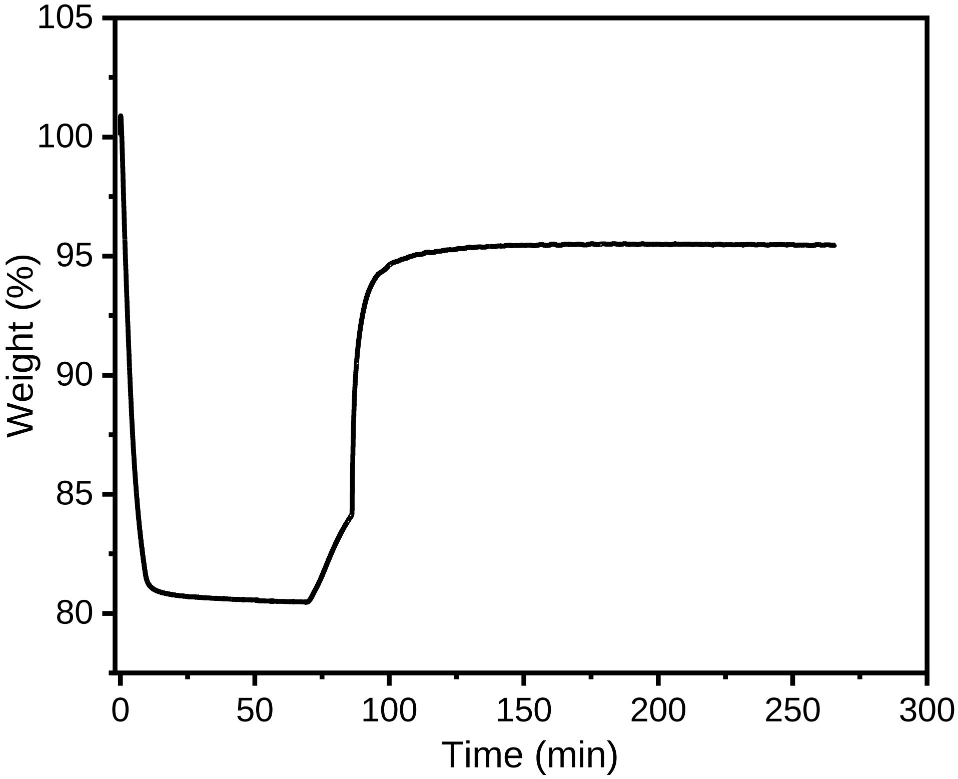

Fig. S10. Desorption: 1-hour at 120°C under N_2, and Adsorption: 3 hours at 30°C under 100% dry CO_2; Cooling rate: 10°C/min

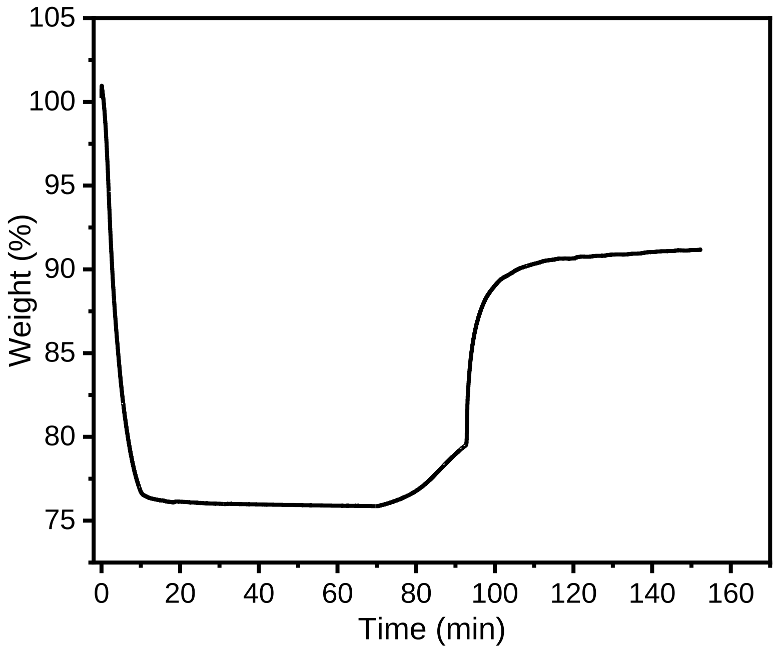

Fig. S11. Desorption: 1-hour 120^oC under N_2, and Adsorption: 1-hour at 30^oC under 100 % dry CO2; Cooling rate: 5^oC/min

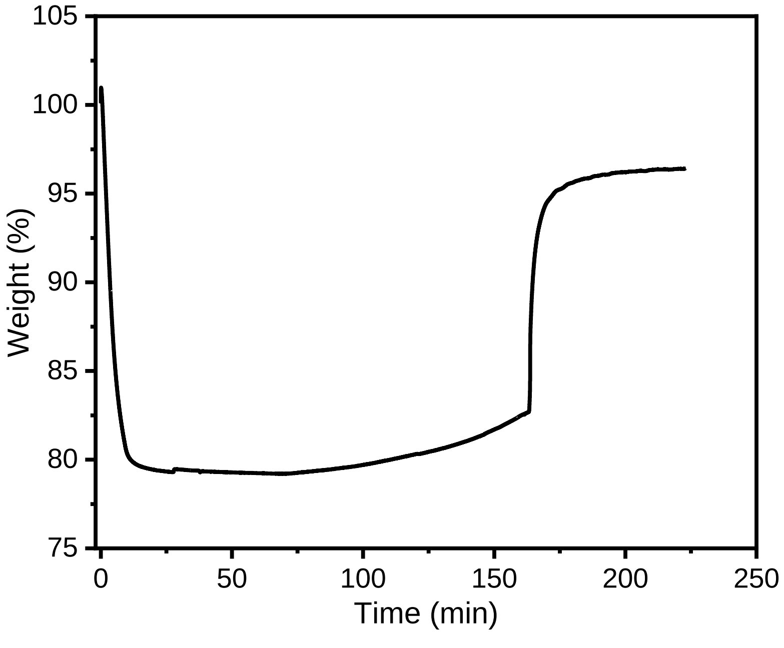

Fig. S12. Desorption: 1-hour at 120^oC under N_2, and Adsorption: 1-hour at 30^oC under CO_2; Cooling rate: 1^oC/min

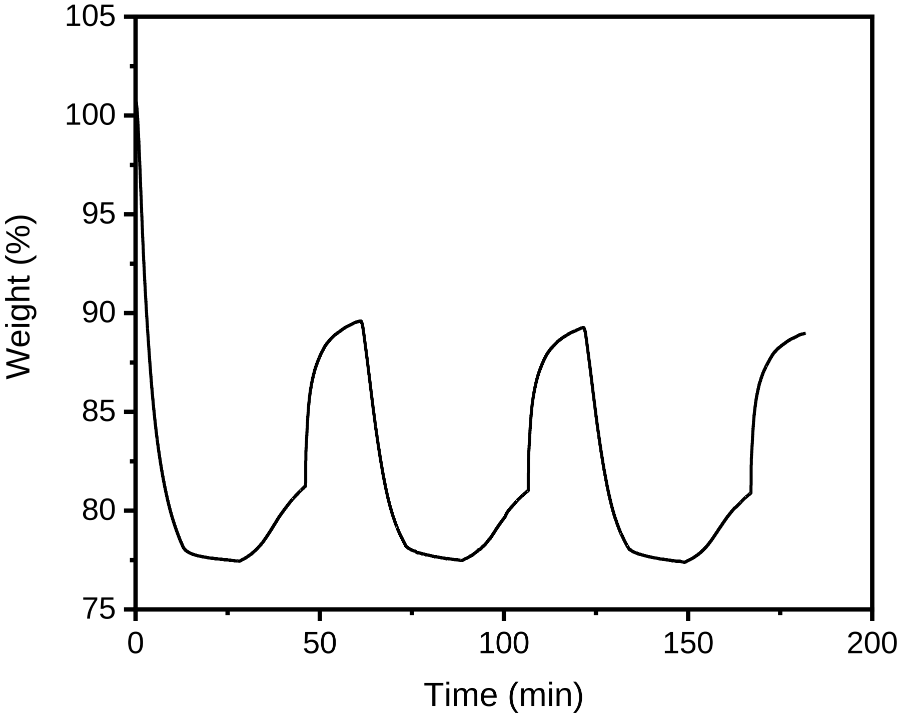

Fig. S13. Desorption: 15 min at 150^oC under N_2, and Adsorption: 15 min at 30^oC under CO_2; Cooling rate: 10^oC/min

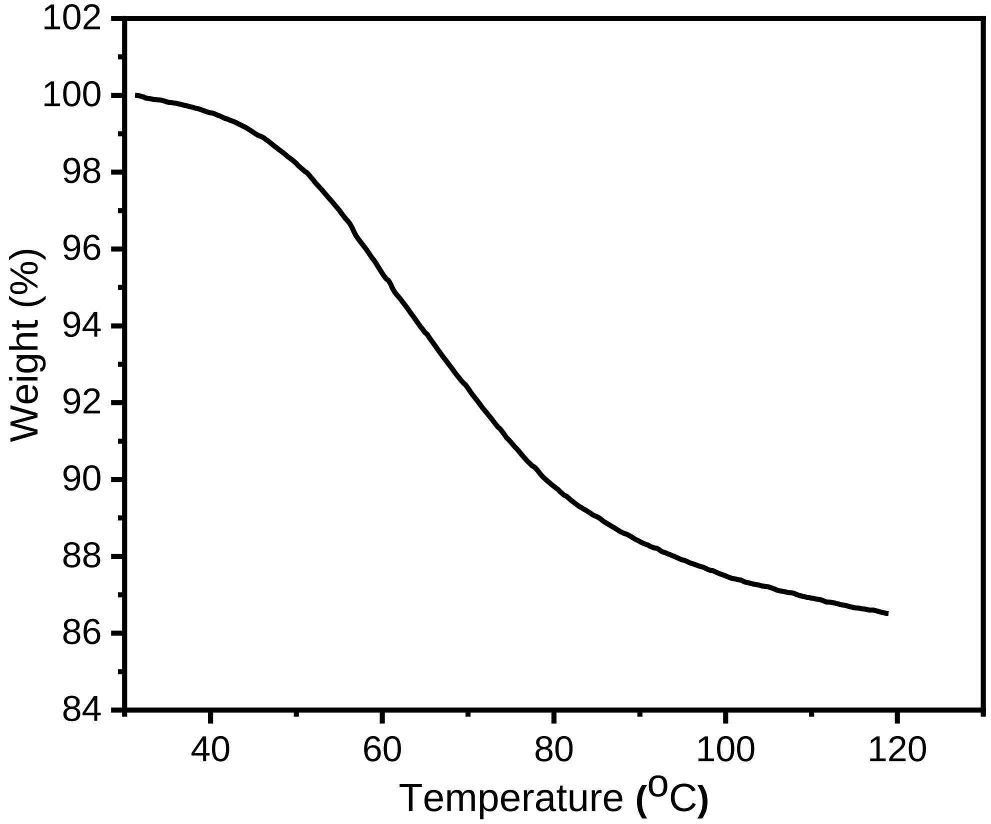

Fig. S14. Thermal desorpion of CO_2 and H_2O from mmen-Mg_2(dobpdc) under He gas after adsorbing humid CO_2 for quantification.

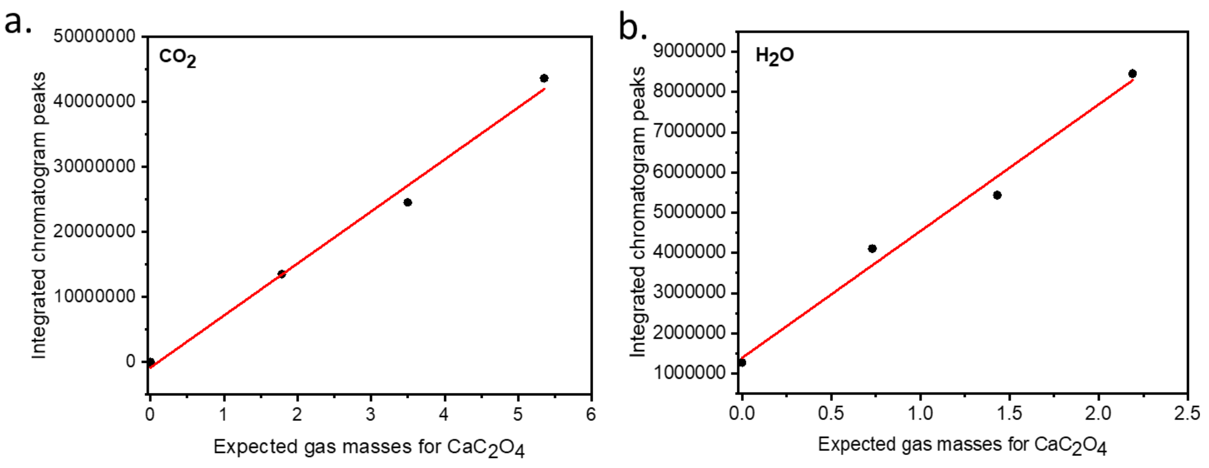

Fig. S15. Calibration curves for (a) CO_2 and (b) H_2O desorbed from standard Calcium oxalate (CaC_2O_4)

References

1. T. M. McDonald, J. A. Mason, X. Kong, E. D. Bloch, D. Gygi, A. Dani, V. Crocellà, F. Giordanino, S. O. Odoh, W. S. Drisdell, B. Vlaisavljevich, A. L. Dzubak, R. Poloni, S. K. Schnell, N. Planas, K. Lee, T. Pascal, L. F. Wan, D. Prendergast, J. B. Neaton, B. Smit, J. B. Kortright, L. Gagliardi, S. Bordiga, J. A. Reimer and J. R. Long, Cooperative insertion of CO2 in diamine-appended metal-organic frameworks, *Nature*, 2015, **519**, 303-308.
2. A. C. Forse, P. J. Milner, J.-H. Lee, H. N. Redfearn, J. Oktawiec, R. L. Siegelman, J. D. Martell, B. Dinakar, L. B. Zasada, M. I. Gonzalez, J. B. Neaton, J. R. Long and J. A. Reimer, Elucidating CO2 Chemisorption in Diamine-Appended Metal–Organic Frameworks, *J. Am. Chem. Soc.*, 2018, **140**, 18016-18031.
